# New Neutron Imaging Facility NIFFLER at Very Low Power Reactor VR-1

**DOI:** 10.3390/jimaging9010015

**Published:** 2023-01-10

**Authors:** Jana Matouskova, Burkhard Schillinger, Lubomir Sklenka

**Affiliations:** 1 Department of Nuclear Reactors, Faculty of Nuclear Sciences and Physical Engineering, Czech Technical University in Prague, V Holesovickach 2, 180 00 Prague, Czech Republic; 2 Heinz Maier-Leibnitz Zentrum (FRM II), Technische Universität München, 85748 Garching, Germany

**Keywords:** neutron radiography, neutron tomography, neutron imaging, very low power research reactor, training reactor VR-1

## Abstract

The paper describes the construction of the neutron imaging facility at the very low-power research reactor VR-1. The training reactor VR-1 is operated by the Czech Technical University in Prague, Czech Republic. It is mainly used for the education of students in the field of nuclear engineering as well as for the training of professionals. Neutron imaging is the new field of VR-1 reactor utilisation currently under development. Extremely low reactor power at the level of 100 W brought many challenges that were necessary to overcome to build and commission a sustainable neutron radiography facility. The paper describes the reactor’s neutron flux verification and the basic concept and design of the neutron imaging instrumentation. The first experimental results were mainly dedicated to testing the detection system for different radial beam port configurations, different L/D ratios, and different exposure times. Preliminary results of neutron radiography and tomography measurements at VR-1 clearly showed the potential of using neutron imaging in low-power reactors such as the VR-1 reactor.

## 1. Introduction

Neutron imaging is most often performed on high-power neutron sources. However, in the last few years, the situation has slowly changed with the development of CCD and CMOS imaging systems. The reasonable price of these systems, together with suitable scintillator screens, has brought new chances for neutron imaging and low-flux neutron sources such as low-power research reactors. However, the low flux is the main but not the only challenge connected with neutron imaging at low-power research reactors. The second serious challenge is related to a specific mode of operation of low-power research reactors. High power reactors are constantly in operation during a cycle of several weeks, and activities can be performed simultaneously. On the other hand, a low-power reactor is usually operated on a one-shift daily basis and usually cannot be used for several activities simultaneously. Therefore, a special reactor time must be dedicated only to neutron imaging. There are several examples in the world of high-quality neutron imaging facilities at medium and low-power research reactors. For example, a highly successful neutron imaging system is a digital neutron computed tomography system at a 250 kW test reactor at the Idaho National Laboratory INL, Idaho Falls, ID, USA [1]. In this context, this study has two main objectives, to demonstrate the feasibility of neutron imaging at low-power reactors, such as the VR-1 reactor, and to extend the range of applications of the VR-1 reactor. Furthermore, as the reactor is now mainly used for education and training, we would like to include neutron radiography as another type of utilization, mainly for educational and demonstration purposes. However, if it is sufficiently improved, it could be possible to use it in research, for example, in the cultural heritage field, industry, etc.

## 2. Neutron Imaging Basic Principle

Neutron imaging is a non-destructive technique used to investigate internal structures and material compositions of optically opaque objects [2]. Other than in X-ray-imaging, where contrast depends on the position in the periodic system and thus the number of electrons in an atom, neutron imaging is also sensitive to many light elements, especially hydrogen, while most metals are penetrated easily. The transmitted neutron beam is detected by—mostly—a camera detector using a cooled scientific camera, a neutron sensitive scintillation screen and a mirror to take the camera out of the direct beam.

This creates a grayscale image or a shadowgraph that contains information about the thickness and material composition of the sample [3]. One of the fundamental quantities used in neutron radiography is the transmission T, which is defined by:

(1) $$T=\frac{I}{I_{0}}$$

where *I* is the intensity of the neutrons that passed through the sample and *I*_0_ is the intensity of neutrons on the sample [4]. The beam intensity is attenuated after passing through the object, and it can be described by the fundamental law of radiation attenuation in a matter (Lambert’s law):

(2) $$I\left(x\right)=I_{0}\cdot \;e^{-\int \Sigma_{tot}d_{x}}$$

where *I*_0_ is the intensity of neutrons incident on the sample, *x* is the path through the object, and Σ*_tot_* is the total macroscopic cross-section [4]. Neutrons can interact with the matter and thereby be removed from the incident beam either by absorption or by a change in direction as they interact with the material in the beam (scattering) [3]. Absorption completely removes neutrons from the beam. Scattered neutrons are, in first approximation, assumed to be also removed from the direct beam, but they may still hit the detector in a place different from the original flight path, thus creating a background of scattered neutrons that produce background noise and blurring. An essential parameter of the neutron imaging facility, which affects the spatial resolution of the resulting image, is the L/D ratio, where *L* is the length between the smallest diameter of the collimator or diaphragm and the sample, and *D* is the smallest diameter of the collimator.

Raw data images obtained from camera software during the measurement need to be processed and analysed in the case the open-source software ImageJ was used. [5] The data processing process includes removing white spots caused by gamma radiation falling directly on the camera chip and normalisation with open beam and dark field images. First, to correct for camera offset and thermal noise, the dark image without a beam must be subtracted from both the projection and open beam images. An open beam image contains the intensity distribution of the beam, and by dividing the result of the dark field corrected projection by the result of the corrected open beam image, the image is normalised to the beam intensity distribution.

## 3. Neutron Imaging on Low Power Research Reactors

According to the International Atomic Energy Agency Research Reactor Database (RRDB), there are 223 [6] research reactors in operation around the world. Of these 223 reactors, 67 [6] research reactors declare the use of neutron imaging and two [6] research reactors that declare the use of neutron imaging are temporarily shut down. The range of potential applications of neutron imaging is primarily limited by reactor power. It is possible to use almost any research reactor with a collimated neutron beam. The main criterium for research reactors for neutron imaging is the intensity of the available neutron beam, which is related to reactor power. Different references present different requirements for neutron intensity/reactor power. The IAEA research reactor utilization matrix from the IAEA Applications of research reactors mentioned no capability for neutron imaging for thermal reactor power of 100 kW and less, and for reactors with a power of less than 1 MW, neutron imaging can be carried out in a limited way, and it is especially suitable for demonstration purposes [7].

Most reactors operating neutron imaging facilities are medium and high power research reactors with reactor power from kilowatts to tens of megawatts, for example, reactor FRM II [8] or high flux reactor at ILL [9]. However, according to IAEA RRDB, there are research reactors with less than 1 kW that declare the use of neutron imaging. These are the Brazilian Argonauta reactor [10], the Japanese UTR KINKI reactor [11] and the Korean AGN-201K reactor [12] (see Table 1.).

Another low-power reactor which recently tested the possibility of using neutron imaging, although this reactor does not declare the use of neutron imaging according to the RRDB, is reactor AKR-2 in Germany [13]. The thermal power of the AKR-2 reactor is two watts [13], and to find a suitable location for the device, a study of L/D and neutron fluxes was performed in [13].

## 4. Training Reactor VR-1

**Figure 1:**
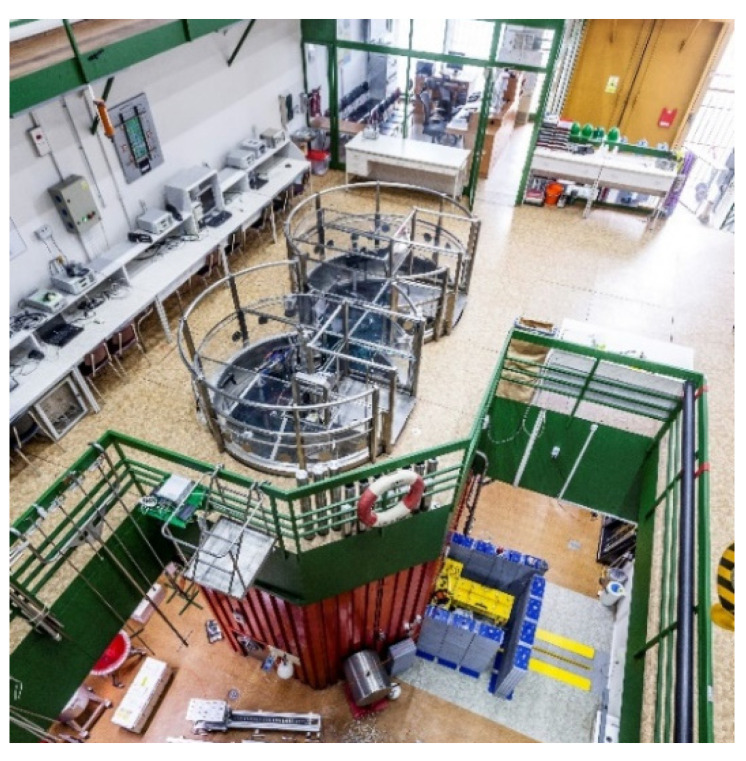
Research reactor VR-1.

The training reactor VR-1 is operated by the Czech Technical University in Prague (CTU). The VR-1 reactor is state-of-the-art experimental instrumentation for the education of students in the field of nuclear engineering from the Czech Republic and abroad. Research and development activities at the reactor are mainly focused on current challenges in nuclear energy development, particularly on the safe operation of nuclear installations, theoretical and experimental reactor physics, nuclear safety and nuclear fuel cycle [14]. Apart from traditional nuclear technology research, the VR-1 reactor is also active in using neutron applications in research, which enables various multidisciplinary research studies that puts together nuclear technology and natural sciences, social sciences and humanities. A photo of the VR-1 training reactor is shown in Figure 1 [14].

The reactor is a pool-type, light water reactor based on low-enriched uranium (19.7% of U235) [14]. The reactor uses IRT-4M-type concentric fuel elements. The nominal thermal power of the reactor is 100 W, which can be increased up to 500 W up to 70 h annually [14]. The reactor is operated at atmospheric pressure at a temperature of about 20 °C. The neutron moderator is demineralised light water, which is also used as a neutron reflector, biological shielding and as a coolant. The experimental equipment of the VR-1 reactor consists of several vertical irradiation tubes, one radial horizontal beam port, one tangential horizontal beam port, shutter and measuring boxes for experiments on a radial beam port, instrumentation for detection of delayed neutrons, instrumentation for bubble boiling simulation, instrumentation for the study of temperature reactivity effects, instrumentation for fast reactivity changes, etc.

## 5. Development of the Neutron Imaging Facility

### 5.1. Neutron Radiography at the VR-1 Reactor

The first step to developing neutron imaging at the training reactor VR-1 was experiments that took place from 2012 to 2016. These experiments were focused on the possibility of using neutron imaging at the VR-1 reactor [15]. It was the development of neutron imaging using photographic film detectors with a converter. Those activities showed the possibility of performing neutron imaging at low-power reactors such as the reactor VR-1. The results of experiments indicated the capability for neutron imaging at the VR-1 reactor even though it is very limited and should be intended primarily for educational and demonstration purposes.

### 5.2. Design and Construction of Experimental Set up

**Figure 2:**
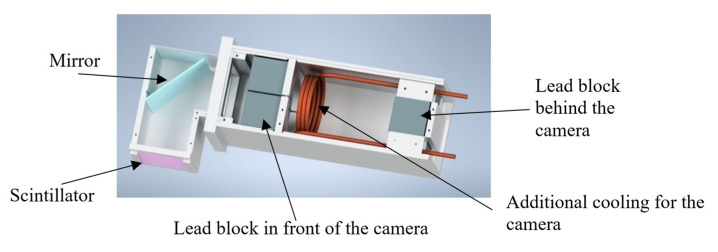
Model of the neutron imaging detection system without the camera.

The experiments from 2013 to 2016 were followed by work in 2020 focusing on neutron imaging using digital imaging methods. In the beginning, a simple neutron radiography detection system was designed in cooperation with the Heinz Maier-Leibnitz Zentrum of the Technical University of Munich [16]. A new neutron imaging facility at the reactor VR-1 called NIFFLER—“Neutron Imaging Facility for Learning and Research”—is based on a cooled CMOS camera combined with a scintillator screen. The CMOS camera is placed inside the special camera box with a lens, additional cooling for the camera and lead shielding. The lead shielding is placed in the camera box to shield the camera from direct gammas that are created at the sample and cause white spots on the chip of the camera. The camera model is the cooled QHY 178 m with 14-bit digitization, 3027 × 2048 pixel array and 2.4 um pixel size [17]. Binning 2 × 2 was used for all measurements. The scintillator screen is made of ^6^LiF/ZnS:Cu. Both 100 µm and 200 µm thickness were tried, but although the 100 µm screen theoretically delivers better resolution, the better detection efficiency and light output of the 200 µm screen provided better images in our low flux. The screen is placed on the scintillator box, which also contains a mirror positioned at an angle of 45°. The mirror ensures that the camera is not placed directly in the beam but perpendicular to it. The field of view of the initial detector system is 6 × 6 cm², for tomography, we used a larger mirror box with 10 × 10 cm². The main parameters of the NIFFLER facility are given in Table 2. The detection system without the camera is shown in Figure 2.

**Figure 3:**
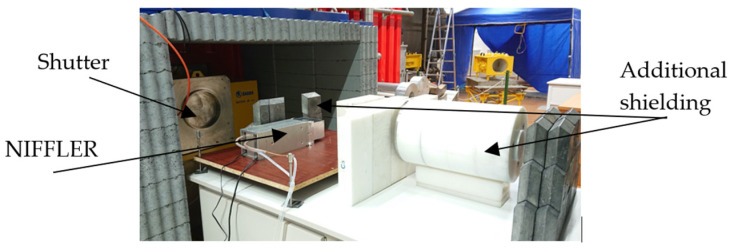
A photo of NIFFLER facility at the reactor VR-1.

A photo of a NIFFLER facility placed at the end of the radial beamline of the training reactor VR-1, with some additional shielding made of lead and borated PE, is shown in Figure 3.

**Figure 4:**
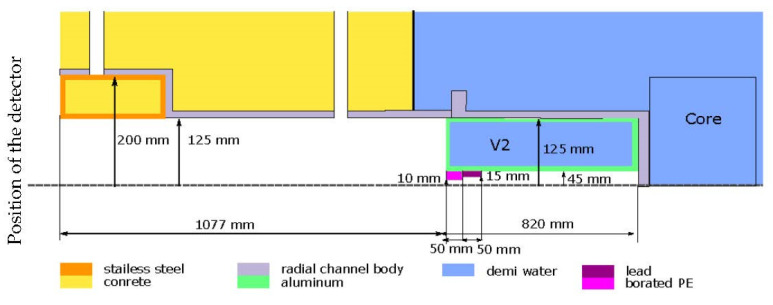
Drawing of the radial beamline with water plug (V2) and pinhole (Pb and PE).

The radial beamline of the reactor VR-1 had to be modified for the purposes of neutron imaging experiments. A hollow aluminum plug containing water with a central channel was first inserted into the radial beamline, limiting the beam from 25 cm to 9 cm (configuration 1). Then, a pinhole was placed at the end of this plug about 80 cm from the reactor core (configuration 2). The pinhole is made of 5 cm lead and 5 cm borated polyethylene, limiting the beam from 9 cm to 2 cm, and it serves to create a beam as parallel as possible and changes the collimation ratio L/D. Figure 4 shows a drawing of the radial beamline with modifications for neutron imaging (V2—cylindrical aluminum plug containing water with an inner diameter of 9 cm, pinhole with an inner diameter of 2 cm).

### 5.3. Neutron Flux Verification

For verification of the thermal neutron flux in the radial beamline of the reactor VR-1, a simulation was performed. The calculation was achieved using the Monte Carlo calculation code SERPENT in version 2.1.31 with a nuclear data library ENDF/B VII.0 [18]. The calculation of thermal neutron flux in the radial beamline was performed at the sample position (190 cm from the core) for two different configurations: Configuration 1—opened radial beamline with a cylindrical aluminum plug containing water (in Figure 4 defined as V2), reducing the beam to 90 mm; Configuration 2—opened radial beamline with a water plug and pinhole (in Figure 4, lead and borated PE).

For verification, the results of the calculation by the serpent calculation code were compared with the measurements with the help of neutron activation analysis (NAA). Neutron activation analysis was performed using gold foils and cadmium covers with Configuration 1 of radial beamline without the pinhole (L/D = 20). The deviation between the calculation in the Serpent calculation code and the neutron activation analysis measurements was around 10%. The results of thermal neutron flux in the radial beamline at the sample position (calculated with Serpent calculation code and measured with NAA) are shown in Table 3.

For both calculations and measurements, a thermal neutron spectrum was verified. With only 1 cm of water between the core and the beam tube nozzle, the neutron spectrum is mostly thermal, with a remaining share of epithermal and fast neutrons, to which the employed scintillation screen is not sensitive. Because of the thermal moderator in the reactor core, there are no cold neutrons in the beam beyond the tail of the Maxwell distribution.

## 6. Results

### 6.1. Neutron Radiography

The first set of neutron imaging measurements was performed to test the detection system with different radial beamline configurations, different L/D and different exposure times. The reactor power for all of these measurements was at the level of 100 Watts.

**Figure 5:**
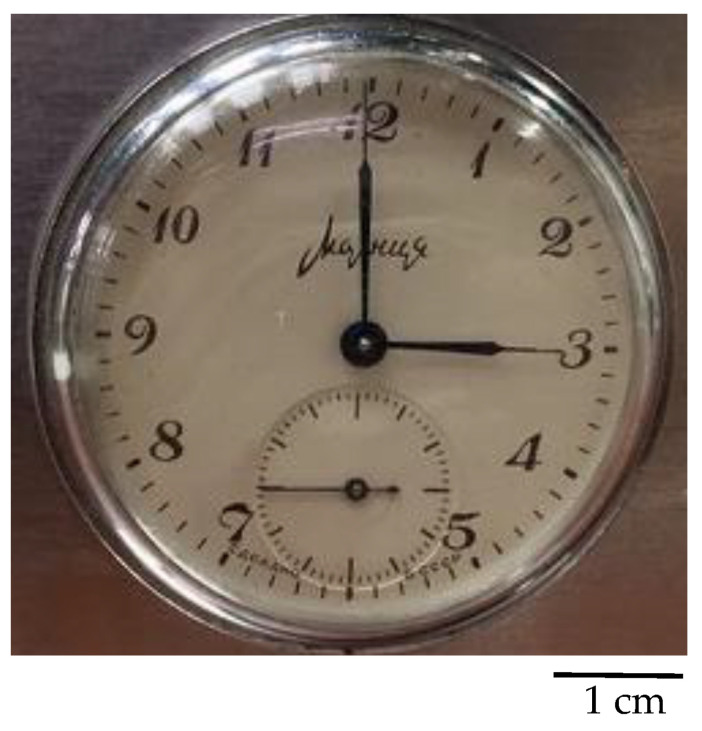
Photo of a pocket watch used as a sample.

**Figure 6:**
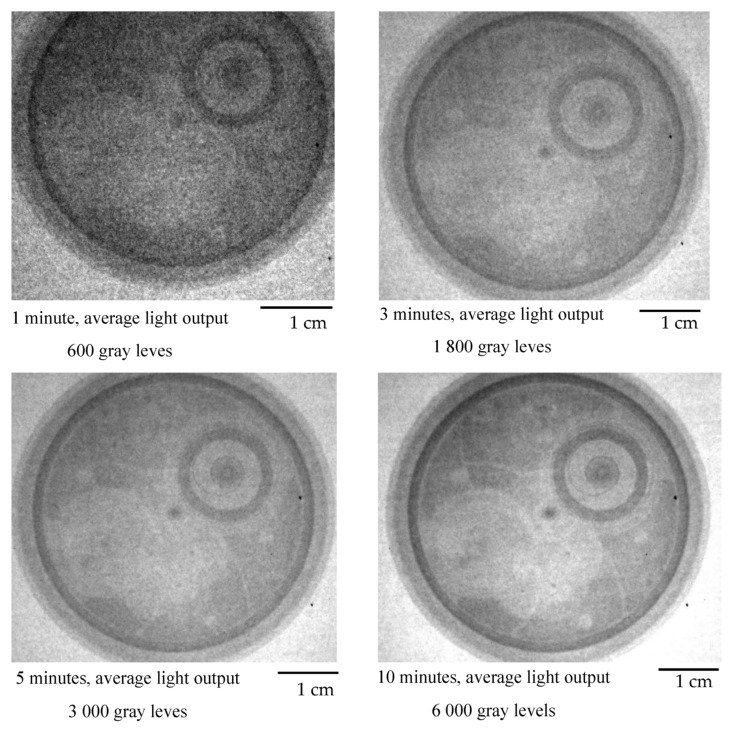
Neutron radiograph of a pocket watch at different exposure times (L/D = 20). Grayscales are stretched for optimal display.

Configuration 1 was used as the first radial beamline configuration. The L/D ratio for this configuration of the radial beamline was 20. Exposure time was chosen step-by-step, and the first radiograph was created after an exposure time of 1 min. Furthermore, measurements were performed for further exposure times of 1–10 min. For the first measurements, a pocket watch was chosen as a sample (Figure 5). The pocket watch was chosen because it has an interesting internal structure and contains different materials. However, its disadvantage is that some parts are very small and therefore difficult to see. Figure 6 shows neutron radiographs of the pocket watch for different exposure times. The grayscales in the images are not identical, and they were stretched for optimum display. Although the 1 min and 3 min exposures look similar in this display, the 3 min exposure clearly has better statistics as can be seen on the sharpness of the central axle of the hands. The sample was placed in direct contact with the screen.

The total fluence per sample for each measurement was also estimated using the SERPENT calculation code. Results of the total fluence are given in Table 4.

**Figure 7:**
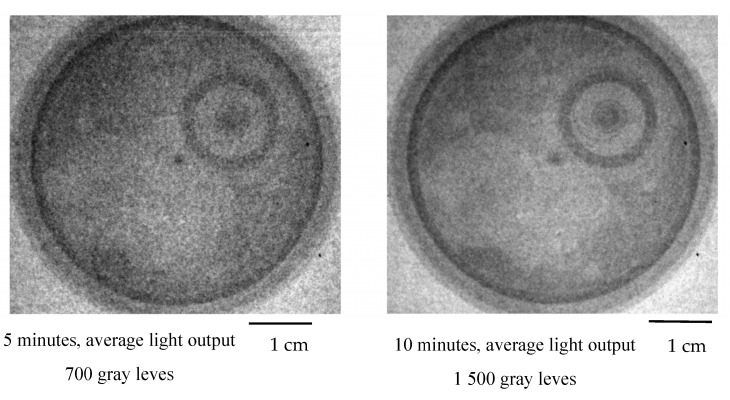
Neutron radiograph of a pocket watch at different exposure times (L/D = 50). Grayscales are stretched for optimal display, and are not the same as Figure 6.

The radial beamline was later modified to Configuration 2. The L/D for this configuration of the radial beamline was 50. Figure 7 shows neutron radiographs of a pocket watch for different exposure times.

The total fluence per sample for this configuration is given in Table 5.

Based on the rule that a higher collimation L/D gives a sharper image, the second set of measurements should bring sharper images. In this case, images suffer from insufficient neutron fluence. Although the L/D is very small for the first set of measurements, it gives a reasonably sharp image because the sample was very thin and placed directly on the scintillation screen.

### 6.2. Neutron Tomography

**Figure 8:**
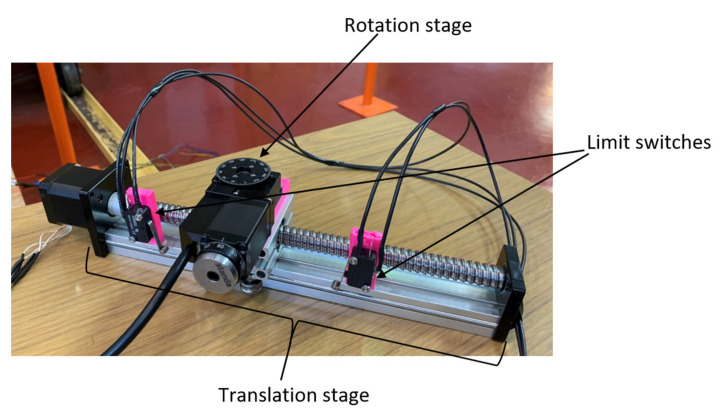
Neutron tomography setup.

A simple and affordable setup was prepared to test the possibility of performing neutron tomography on a reactor with such low power. The setup is based on a simple rotation stage, translation/linear stage and a controller. This controller consisted of a Raspberry Pi and a GERTBOT motor controller [19], which has now been replaced by a Waveshare HAT controller [20] since the original board went out of production. The tomography sequence is composed of several steps, the first step is to take dark field images with a closed beam shutter, and the next step is to move the sample out of the field of view and take open beam images. Both are necessary for image normalization, which is achieved by subtracting the dark field for camera offset and dark current, divided by the open beam to correct for beam profile. The last step of the tomography sequence is to move the sample back into the field of view and perform tomography measurements. This tomography sequence is controlled by a Python script on the Raspberry Pi [21]. A photo of the tomography setup is given in Figure 8.

**Figure 9:**
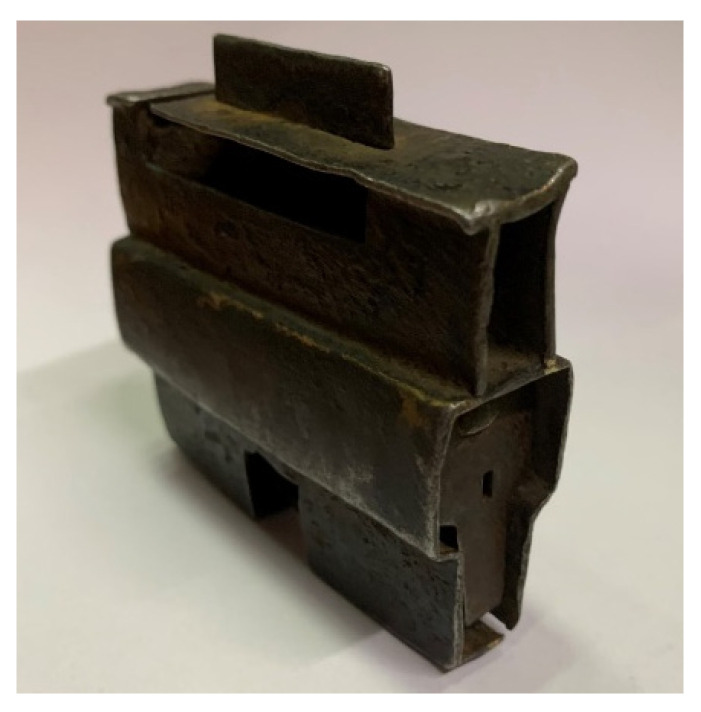
Ancient Tibetan lock used as a sample.

Parameters for the neutron tomography test were chosen based on the neutron radiography results. The measurement was performed in the configuration 2 of the radial beamline with the L/D = 50. The power of the reactor was increased from 100 to 500 watts. Thus, the thermal neutron flux at the sample position was also five times higher than in the case of previous measurements, i.e., 1.75·× 10^5^ $n/{\mathrm{cm}}^{2}s$. The exposure time was chosen as 4 min per projection. The number of projections was primarily limited by the operating time of the reactor (the VR-1 reactor is operated in daily shifts and shut down overnight). Therefore, the longest time possible was used for the neutron tomography measurement, i.e., 12 h of reactor operation, which corresponded to 155 projections, five open beam images and five dark field images. An ancient Tibetan lock was chosen as a sample for the first test tomography, primarily for its interesting internal structure and the possible future use of neutron imaging on the VR-1 reactor to examine cultural heritage. The sample was placed about 5 cm from the scintillator screen. Figure 9 shows a photo of the ancient Tibetan lock. Due to the use of a larger sample, the field of view was also increased from the original 6 × 6 cm to 10 × 10 cm. For this purpose, only the scintillation/mirror box was replaced by a box with larger dimensions, and it was not necessary to change the entire detection system.

**Figure 10:**
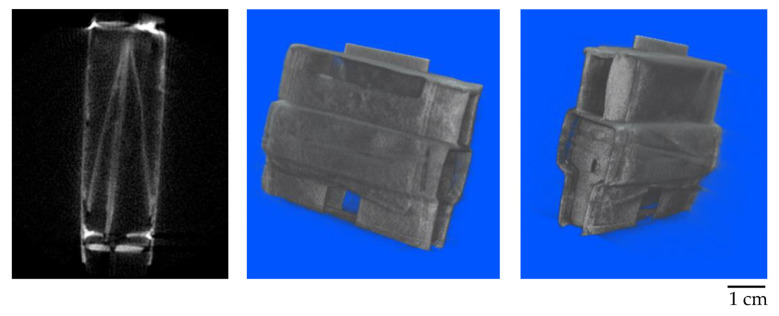
Results of first neutron computed tomography at 500 W, a tomographic slice and 3D views.

Two software programs were used to process the measured data: Octopus by Inside Matters [22] for reconstruction and VGStudio by Volume Graphics [23] for volume rendering. Figure 9 and Figure 10 show the results of the first neutron computed tomography performed on a 500 W reactor. Due to bad neutron statistics, the tomographic reconstruction is noisy but sufficient to reconstruct the object.

## 7. Discussion and Conclusions

This study focused on neutron imaging at the very low power reactor VR-1 using digital imaging techniques. A radial beamline of the reactor and a simple neutron imaging detection system designed in cooperation with Heinz Maier-Leibnitz Zentrum of the Technical University of Munich were used for these measurements. One task for the first neutron radiography measurements at the training reactor VR-1 was to determine the absolute thermal neutron flux at different positions in the radial beamline, especially at the sample position. Detailed flux verification was performed using the calculation code SERPENT. Finally, the calculation of neutron flux was compared to the results of the neutron activation analysis. The first set of neutron imaging measurements was performed to test the detection system for different radial beamline configurations, different L/D and different exposure times. Furthermore, the first neutron computed tomography was performed on a very low-power reactor at the level of 500 W.

Results of neutron imaging measurements at VR-1 showed the potential of performing neutron imaging at low-power reactors such as the VR-1 reactor. The two main objectives of this study were to demonstrate the feasibility of neutron imaging at low-power reactors, such as the VR-1 reactor, and to extend the range of application of the VR-1 reactor. The NIFFLER neutron imaging facility at the VR-1 reactor will be further modified and improved to achieve the best possible results. Future work will primarily focus on modifications of the neutron spectrum and the modification of the beamline in order to optimise the L/D and the neutron flux at the sample position.

## Figures and Tables

**Table 1:** Low power research reactors using neutron radiography according to RRDB [5].

Country	Facility/Type	Power (Watts)
Brazil	Argonauta/argonaut	200
Japan	UTR KINKI/argonaut	1
Korea	AGN-201K/homogeneous	10

**Table 2:** Main parameters of the NIFFLER neutron imaging facility.

Camera type	CMOS (QHY 178 m cooled, 3072 × 2048 pixel)
Camera digitization	14-bit
Lens type	25 mm C mount
Scintillator screen	6LiF/ZnS:Cu
Field of view Effective pixel size	6 × 6 cm² or 10 × 10 cm² 39 µm or 65 µm (2 × 2 bin)

**Table 3:** Calculated and measured thermal neutron flux in different configurations of radial beamline.

Configuration	Verification	$\mathrm{Thermal}\;\mathrm{Flux}\;\phi_{th}\left(n/cm^{2}s\right)$
Without the pinhole (L/D = 20)	SERPENT	2 × 10^5^
With the pinhole (L/D = 50)		3.5 × 10^4^
Without the pinhole (L/D = 20)	NAA	1.85 × 10^5^

**Table 4:** The total fluence per sample for different exposure times (L/D = 20).

Exposure Time (Minutes)	$\mathrm{Fluence}\;\left(n/cm^{2}\right)$
1	1.2 × 10^7^
3	3.6 × 10^7^
5	6 × 10^7^
10	12 × 10^7^

**Table 5:** The total fluence per sample for different exposure times (L/D = 50).

Exposure Time (Minutes)	$\mathrm{Fluence}\;\left(n/cm^{2}\right)$
5	1.05 × 10^7^
10	2.1 × 10^7^

## Data Availability

Not applicable.
